# Amazon rainforest rodents (Proechimys) are resistant to post-stroke epilepsy

**DOI:** 10.1038/s41598-021-96235-5

**Published:** 2021-08-18

**Authors:** Nancy N. Ortiz-Villatoro, Selvin Z. Reyes-Garcia, Leandro Freitas, Laís D. Rodrigues, Luiz E. C. Santos, Jean Faber, Esper A. Cavalheiro, Josef Finsterer, Fulvio A. Scorza, Antônio C. G. de Almeida, Carla A. Scorza

**Affiliations:** 1 Disciplina de Neurociência, Departamento de Neurologia/Neurocirurgia, Escola Paulista de Medicina/Universidade Federal de São Paulo (EPM/UNIFESP), São Paulo, 04039-032 Brazil; 2 Posgrado de Neurología, Facultad de Ciencias Médicas, Universidad Nacional Autónoma de Honduras, Tegucigalpa, Honduras; 3 Neurociência Experimental e Computacional, Universidade Federal São João Del-Rey, São João del-Rei, Brazil; 4 Krankenanstalt Rudolfstiftung, Mersserli Institute, Vienna, Austria

**Keywords:** Neuroscience, Neurology

## Abstract

There are no clinical interventions to prevent post-injury epilepsy, a common and devastating outcome after brain insults. Epileptogenic events that run from brain injury to epilepsy are poorly understood. Previous studies in our laboratory suggested Proechimys, an exotic Amazonian rodent, as resistant to acquired epilepsy development in post-status epilepticus models. The present comparative study was conducted to assess (1) stroke-related brain responses 24-h and 30 days after cortical photothrombosis and (2) post-stroke epilepsy between Proechimys rodents and Wistar rats, a traditional animal used for laboratory research. Proechimys group showed smaller volume of ischemic infarction and lesser glial activation than Wistar group. In contrast to Wistar rats, post-stroke decreased levels of pro-inflammatory cytokines and increased levels of anti-inflammatory mediators and growth factors were found in Proechimys. Electrophysiological signaling changes assessed by cortical spreading depression, in vitro and in vivo, showed that Wistar’s brain is most severely affected by stroke. Chronic electrocorticographic recordings showed that injury did not lead to epilepsy in Proechimys whereas 88% of the Wistar rats developed post-stroke epilepsy. Science gains insights from comparative studies on diverse species. Proechimys rodents proved to be a useful animal model to study antiepileptogenic mechanisms after brain insults and complement conventional animal models.

## Introduction

Brain injury is a dreadful threat to animals and the capacity for repairing wounded tissue is essential to vertebrate neurobiology. Annually, nearly 15 million people worldwide experience a stroke and approximately 15% of them develop epilepsy ^1^. Despite numerous studies, there is no intervention to prevent post-stroke epilepsy, a complication that is frequently resistant to existing preventive antiepileptic pharmacotherapy ^2^. Epileptogenesis is the process by which brain injury-initiated maladaptive alterations lead to the first spontaneous seizure, the onset of epilepsy. There is a large gap in our understanding of the underlying multifaceted and complex pathophysiological mechanisms of epileptogenesis and how maladaptive plasticity ends up in epilepsy is a topic of major interest. Solid evidence from numerous clinical and animal studies has suggested that the main hallmarks of the brain dynamic response to ischemic stroke include damage to neurons, glial reactivity, release of inflammatory cytokines and growth factors, and alterations in neural excitability, exposing the brain to an environmental calamity, eventually leading to epileptogenesis ^3^. Evolutionary demands seem to have molded incontestable elemental tissue responses to injury that are common across different organs, particularly the brain ^4^. Mounting evidence points out that treating brain disorders requires a profound understanding of brain-specific multicellular responses to injury. It is challenging to explore the intrinsic mechanisms underlying post-stroke brain lesion in mammals and the use of non-traditional animal models may improve insight into epileptogenesis. A comparative approach allows the analysis of similarities and unique characteristics of remarkably different animal species to better understand the deleterious and reparative mechanisms of recovery following brain injury. Previous findings of our laboratory showed that *Proechimys* rodents are resistant to epileptogenesis in traditional models of temporal lobe epilepsy ^5^. Accumulating evidence suggests that inflammation constitutes a decisive piece in the evolution of post-injury brain repair, and we have shown recently that *status epilepticus* does not trigger upregulation of inflammatory mediators in the *Proechimys*’ brain ^6^. Each animal species is adapted to the conditions of the environment in which it evolved; *Proechimys* are small terrestrial rodents of the Amazon rainforest that play crucial roles in tropical zoonotic diseases and are natural hosts of infectious agents ^7^. Nevertheless, they appear to have an effective immune system as they rarely develop diseases ^8^. The present study examined the multifaceted brain responses to photothrombotic cortical ischemia in *Proechimys* in comparison to Wistar rats. We used comparative approaches between these two animal species to unravel the dynamics of brain responses to stroke, at 24-h and 30 days after injury.

## Results

### Photothrombotic stroke produces smaller lesion volume in Proechimys brain

**Figure 1:**
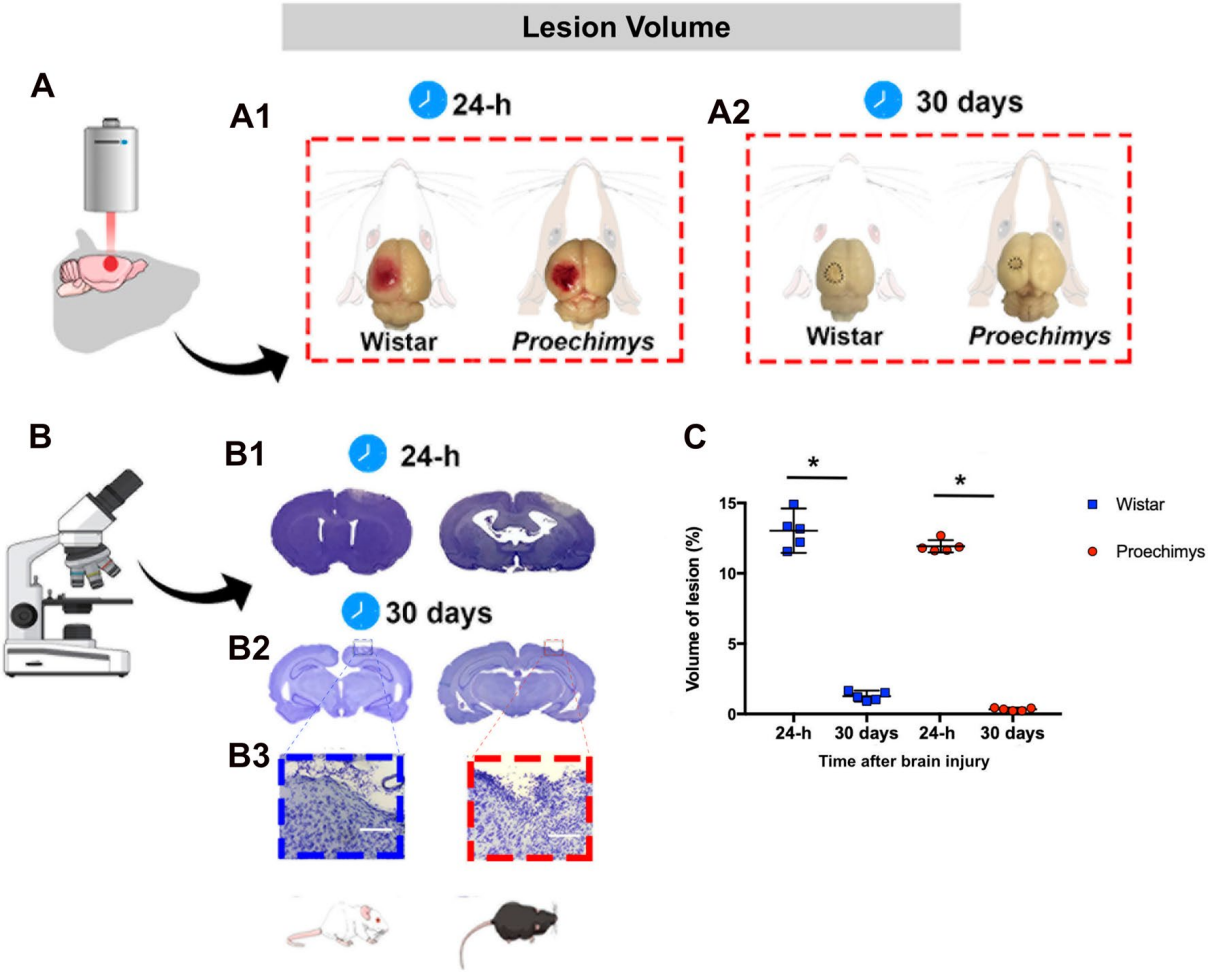
Infarct volume sizes in the Amazon rodents and Wistar rats 24-h and 30 days following photothrombotic ischemia. (**A**) Macroscopic view of the ischemic lesion after ischemic stroke. Wistar left side and Proechimys right side. (**B**) Representative Nissl-stained brain sections showing infarct areas at 24-h (**B1**) and 30 days (**B2**) after stroke. In (**B3**) shows a magnification of × 10 in the infarct area. Wistar left side and Proechimys right side. (**C**) Brain volumes were estimated using the Cavaliere method from Nissl slices. Graphs show the comparisons of the infarct size quantification expressed as a percentage between 1 and 30 days after ischemic stroke in each animal species with 95% CI. Note the lower infarct volumes in both animals at 30 days after ischemic stroke*.* Nonparametric test Mann–Whitney U = 0 *p ≤ 0.05 Wistar, 24-h; 30 days (n = 5). Nonparametric test Mann–Whitney U = 0 *p ≤ 0.05 *Proechimys*, 24-h; 30 days (n = 5). Graphs are represented as median with 95% CI. Figure was created in the Mind the Graph platform http://www.mindthegraph.com under Creative Commons License CC community as “attribution share-alike4.0 licensing” https://creativecommons.org/licenses/by/4.0/.

The cresyl violet staining allowed the detection of unstained damaged areas after stroke. Focal ischemia induced by photothrombosis resulted in macroscopically visible brain injury in both Proechimys and Wistar groups assessed at 24-h after stroke (Fig. 1A1). The relative lesion size expressed as a percentage of the total ipsilateral cortex was 12% in Proechimys and 13% in Wistar rats (Fig. 1,B1,C). At 30 days, lesion volumes were highly reduced in both animal species. The infarct size at 30 days after photothrombotic damage was 0.5% in Proechimys and 3% in Wistar rats (Fig. 1,A1,A2,B1,B2,B3,C). Temporal evolution of brain damage revealed that ischemic damage resulted in much smaller infarct lesion in Proechimys.

### Photothrombotic stroke leads to lower glial cell activation in *Proechimys* rodents

**Figure 2:**
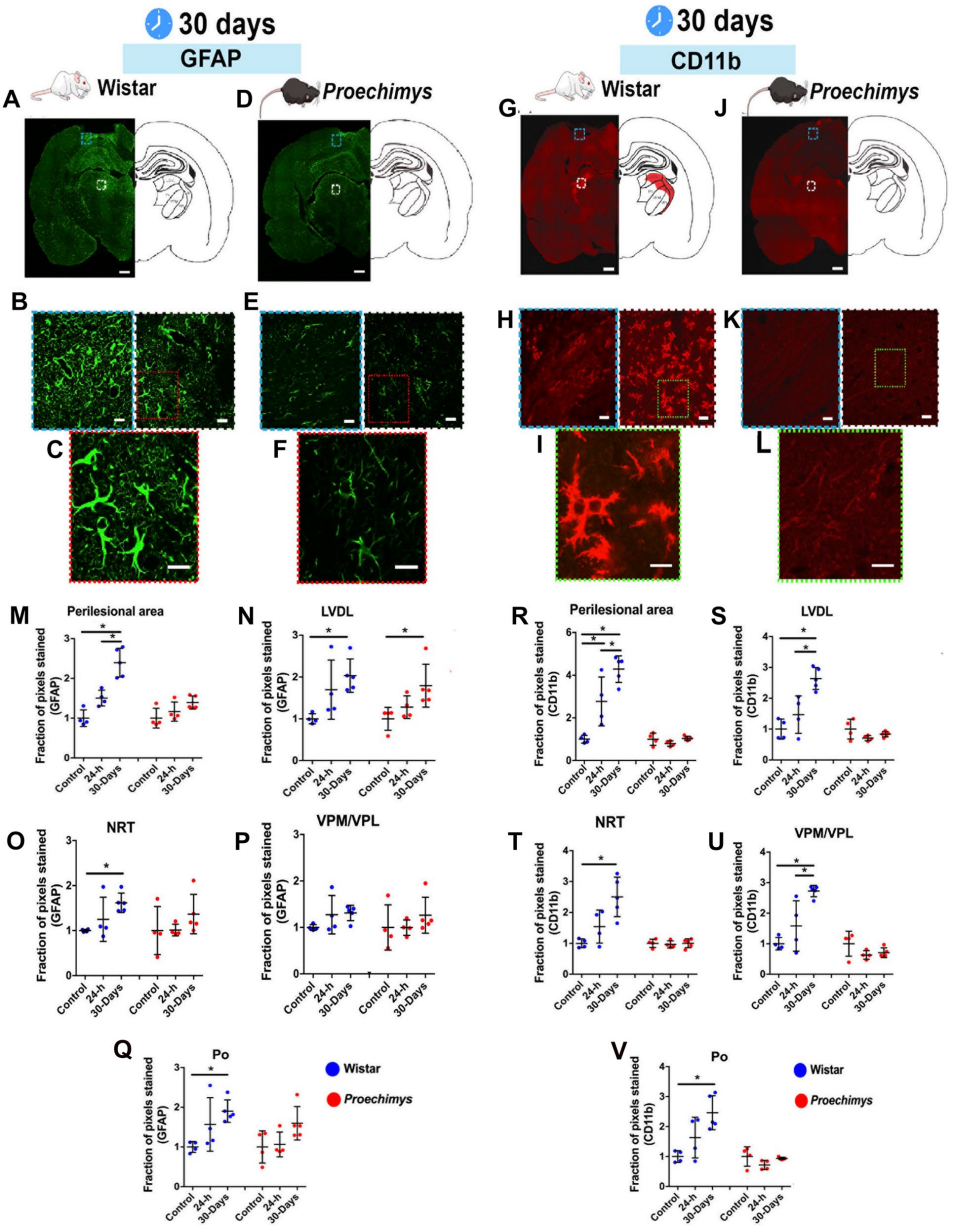
GFAP and CD11b expression after photothrombotic stroke. (**A–L**) Representative images of immunofluorescence staining of glial cells at 30 days folalowing cortical ischemia**.** GFAP (green) (Wistar (**A–C**); *Proechimys* (**D–F**)) and CD11b (red) (Wistar (**G–I**) *Proechimys* (**J–L**)). (**B,E**) Insets show cortical perilesional area (blue boxes, left side) and LDVL thalamic nucleus (white boxes, right side) in both animal species, Wistar (**B**) and Proechimys (**E**). (**C**,**F**) Higher magnification views show thalamic astrocytes. Note the marked hypertrophic phenotype of thalamic astrocytes in Wistar. (**H,K**) Insets show CD11b in the cortical perilesional area (blue boxes, left side) and LDVL thalamic nucleus (white boxes, right side) in both animal species, Wistar (**H**) and Proechimys (**K**). (**I,L**) Higher magnification shows reactive microglia in the thalamus of Wistar rats with retracted processes and enlarged cell bodies (**I**) and the absence of CD11b in *Proechimys* (**L**). (**M**–**V**) Statistical analysis of glial cells immunostaining among groups: control *vs* 24-h, control *vs* 30 days, 24-h *vs* 30 days. Each animal species was divided as follows: control (n = 4); 24-h (n = 4) and 30-day (n = 5). (**M**–**Q**) GFAP and (**R**–**V**) CD11b. In (**A,D**), the white bars indicate 1 mm, in (**G,J**), 1.3 mm. In (**B,E,H,K**), the white bars in the white and blue squares indicate, 20 µm. The red boxes (**C,F**) indicate white bars with 10 µm and the green boxes (**I,L**) indicate white bars with 8 µm. Data are presented as means ± SD. For statistical analysis ANOVA One-way was used. Multiple comparisons were performed by Tukey’s post hoc test *p ≤ 0.05.

In our work, glial cell activation was assessed at 24-h and 30 days after stroke using GFAP (glial fibrillary acidic protein) as a marker of astrocytes and CD11b for microglia/macrophage activation (Fig. 2). In Wistar rats, reactive astrocytic cells were not observed at 24-h after photothrombosis, as previously described ^9^, but significant reactive astrocytes were found at 30 days post-stroke in the perilesional area, as indicated by the number of pixels counted, which reflects the expansion of the area occupied by reactive astrocytes (F = 29.29, p < 0.0001. Tukey’s post hoc test showed a difference between control vs 30 days and 24-h vs 30 days, Fig. 2M). Studies have suggested that several thalamic nuclei contribute to post-injury epileptogenesis ^10,11^. Most specifically, the appearance of increased intra-thalamic excitability restricted to gliotic areas shortly after focal cortical infarct contributing to stroke-induced epileptogenesis in rats has been reported ^11^. Since Paz et al. showed that cortical photothrombosis resulted in spontaneous ictal activity only in the gliotic thalamus, we chose to analyze glial cells specifically in the thalamic nuclei following stroke, (LDVL (Lateral dorsal ventrolateral): F = 5.52, *p = 0.0242, Fig. 2N; NRT (Reticular thalamic nuclei): F = 4.792, *p = 0.0347, Fig. 2O; Po (Pulvinar): F = 5.232, *p = 0.0279, Fig. 2Q. Tukey’s post hoc test showed a difference between control vs 30 days after stroke in all thalamic nuclei). In *Proechimys*, reactive astrocytes were only found in the LDVL thalamic nucleus at 30 days after stroke (F = 4.904 *p = 0.0328 (Fig. 2N)).

**Figure 3:**
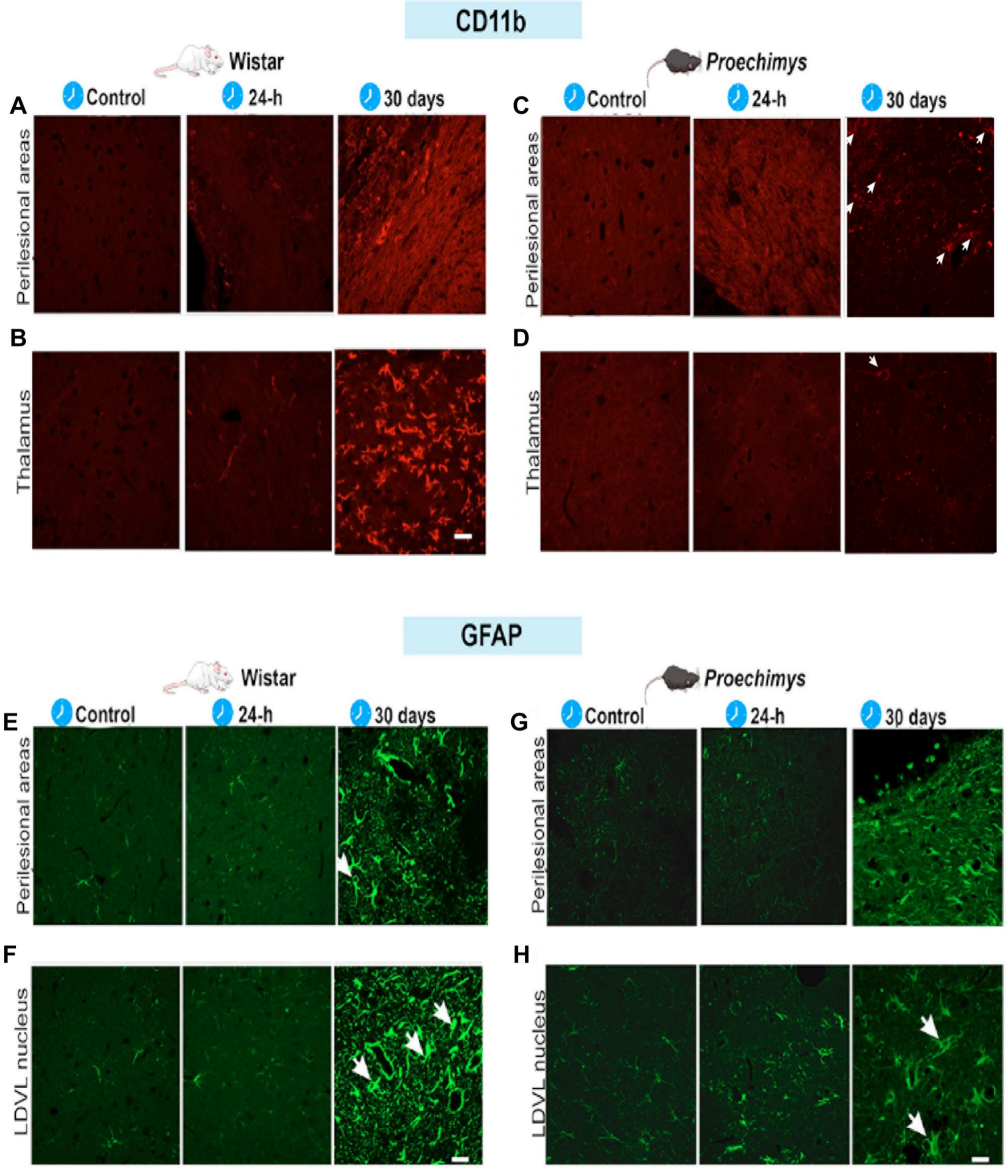
CD11b and GFAP immunofluorescences staining in the perilesional area and thalamus in Wistar and *Proechimys*. (**A**–**D**) Representative immunostaining images of CD11b in the perilesional area (**A**,**C**) and thalamus (**B**,**D**). Note the absence of CD11b positivity in *Proechimys* and increased CD11b expression at 30 days after photothrombosis in both brain regions of Wistar rats. (**E**–**H**) Representative images of GFAP in the perilesional area (**E**,**G**) and LDVL thalamic nucleus (**F**,**H**). In Wistar, increased expression of GFAP was found at 30 days in the perilesional area (**E**) and in several thalamic nuclei; (**F**) shows the LDVL nucleus. In *Proechimys*, increased expression of GFAP was solely found in the LDVL nucleus at 30 days after stroke. Scale bars 20 µm.

CD11b-positive microglia presented morphological changes suggestive of activated state (de-ramification and increased soma size) (Fig. 3) and CD11b-immunoreactivity was higher in the Wistar rats in the perilesional area when comparing 24-h and 30 days groups with controls (F = 21.44 *p = 0.0002 (Fig. 2R)). In thalamic nuclei the highest CD11b-immunoreactivity at 30 days post-stroke was observed in LDVL (F = 16.98 *p = 0.0006 (Fig. 2S)); NRT (F = 23.72 *p < 0.0001 (Fig. 2T)); VPM/VPL (Ventral posteromedial/ Ventral posterolateral) (F = 15.16 *p = 0.0009 (Fig. 2U)); Po (F = 8.674 *p = 0.0065 (Fig. 2V)). CD11b immunostaining was absent in all groups of *Proechimys* rodents (Fig. 3C,D).

### Distinctive profile of neutrophin and cytokine expression produced by brain tissue injury in the Amazon rodents

In the photothrombotic model, the laser beam irradiates the photosensitizing dye that destroys the cortical microvasculature, elevating the levels of inflammatory cytokines and growth factors, playing a fundamental role in the pathophysiology of ischemic stroke ^9^. Increasing attention has been devoted to the variations in the expression of these factors that can act parallel or sequentially during post-injury brain recovery.

**Figure 4:**
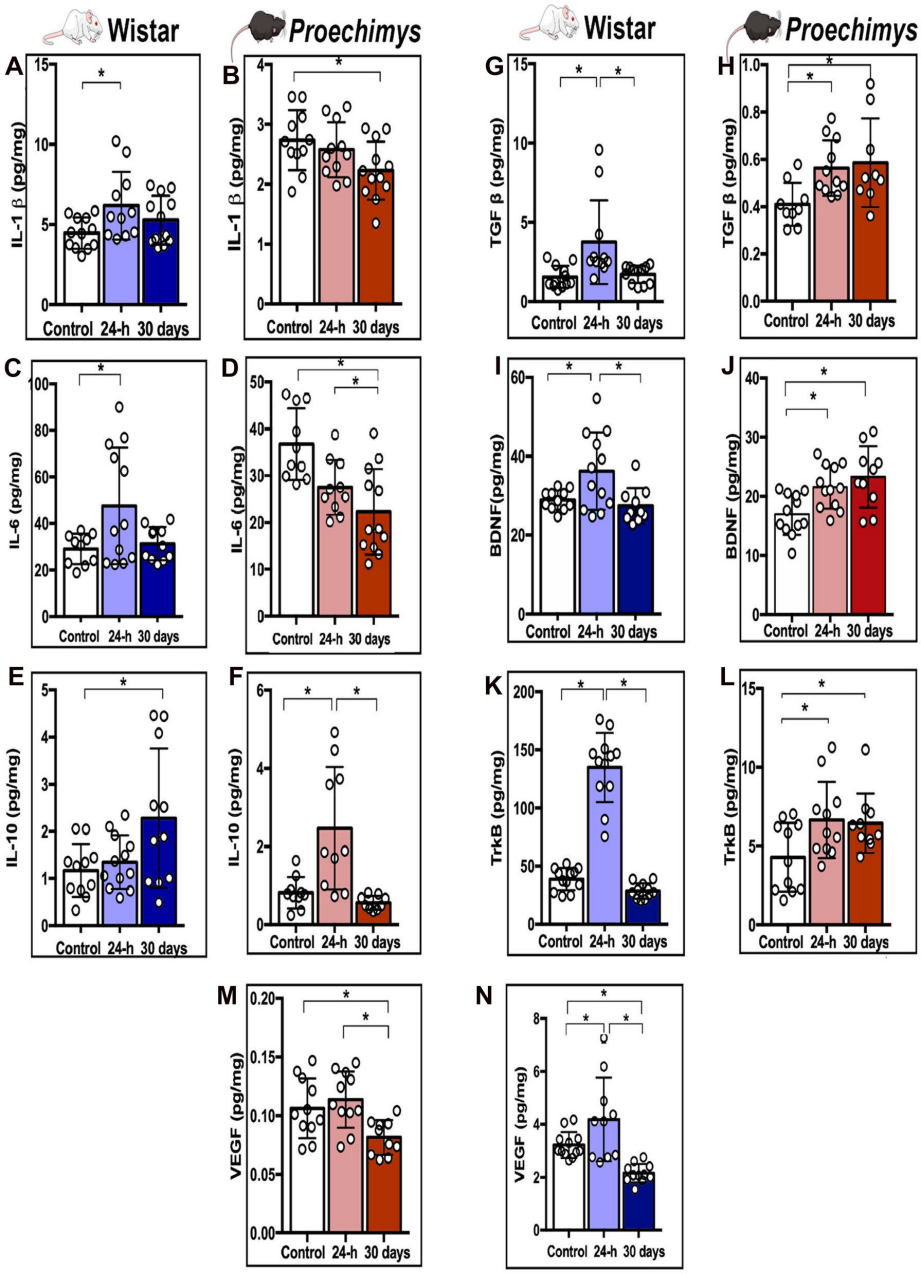
Brain levels of inflammatory cytokines/growth factors after photothrombosis stroke. Assessment of cytokines and chemokines analysis using multiplex assay ELISA. Bar graphs show quantification of protein levels cytokines/growth factors in the ischemic brain tissue 24-h and 30 days after photothrombosis stroke vs. control. One-way ANOVA followed by Tukey test. *p < 0.05. Graph bars show mean ± SD. (**A,B**) IL-1β levels Wistar (**A**) and *Proechimys* groups (**B**). (**C**,**D**) IL-6 levels in Wistar (**C**) and *Proechimys* group (**D**). (**E**,**F**) IL-10 levels in Wistar group (**E**) and *Proechimys* group (**F**). (**G**,**H**) TGF-β levels in Wistar (**G**) and *Proechimys* group (**H**). (**I**,**J**) BDNF levels in Wistar (**I**) and *Proechimys* group (**J**). (**K**,**L**) TrkB levels in Wistar group (**K**) and *Proechimys* group (**L**). (**N**,**M**) VEGF levels in Wistar group (**N**) and *Proechimys* group (**M**).

Here, we sought to examine the concentrations of inflammatory cytokines (IL-1β, IL-6, and IL-10) and growth factors (VEGF, BDNF/TrkB and TGF-β) 24-h and 30 days after ischemic stroke. We found that IL-1β and IL-6 levels were increased solely 24-h after stroke in Wistar rats (F = 3.351 *p < 0.05; F = 4.455 *p < 0.05 respectively) (Fig. 4A,C). *Proechimys* showed unchanged IL-1β content at 24-h, but these levels were decreased at 30 days (F = 3.342 *p < 0.05) (Fig. 4B). *Proechimys* exhibited decreased IL-6 expression at 24-h and 30 days when compared with control group (F = 9.526 *p < 0.05) (Fig. 4D).

In Wistar rats, similar IL-10 levels were observed in controls and the 24-h group, but IL-10 expression was increased at 30 days (F = 4.31 *p ≤ 0.05) (Fig. 4E). On the contrary, similar IL-10 levels were observed in controls and in *Proechimys* at 30 days, but IL-10 expression was increased at 24-h (F = 11.35 *p ≤ 0.05) (Fig. 4F).

In Wistar rats, TGF-β and BDNF/TrkB levels were increased solely 24-h after photothrombotic stroke (TGF-β (F = 6.864 *p ≤ 0.05) (Fig. 4G); BDNF (F = 5.734 *p ≤ 0.05) (Fig. 4I) and TrkB (F = 115.3 *p ≤ 0.05) (Fig. 4K)). In *Proechimys,* increased levels of TGF-β and BDNF/TrkB were found at 24-h and 30 days after injury (TGF-β (F = 4.495 *p ≤ 0.05) (Fig. 4H); BDNF (F = 7.133 *p ≤ 0.05) (Fig. 4J) and TrkB (F = 115.3 *p ≤ 0.05) (Fig. 4L)).

In comparison to baseline levels, enhanced levels of VEGF were detected at 24-h and a decrease was found at 30 days in Wistar rats (F = 12.34 *p ≤ 0.05) (Fig. 4N). In *Proechimys*, unchanged VEGF expression was observed at 24-h but decreased levels were observed at 30 days as compared with controls and the 24-h groups (F = 6.091 *p ≤ 0.05) (Fig. 4M).

In summary, in Wistar rats, IL-1β, IL-6, TGF-β, BDNF/TrkB and VEGF levels were increased at 24-h after stroke, IL-10 was increased at 30 days. In *Proechimys* rodents, IL-1β increased at 30 days, IL-6, TGF-β and BDNF/TrkB increased at 24-h and 30 days, and IL-10 increased at 24-h. However, IL-6 (at 24-h and 30 days) and VEGF levels (at 30 days) were decreased.

A considerable body of evidence has reported the increased levels of inflammatory/trophic factors shortly after photothrombosis ^9^ but *Proechimys*’ data did not support these previous findings. In the dynamic balance of endogenous factors released in response to stroke, *Proechimys* seems to prioritize the potential protective molecules during recovery from a brain lesion.

### Cortical stroke did not result in spontaneous ictal activity in *Proechimys* rodents

**Figure 5:**
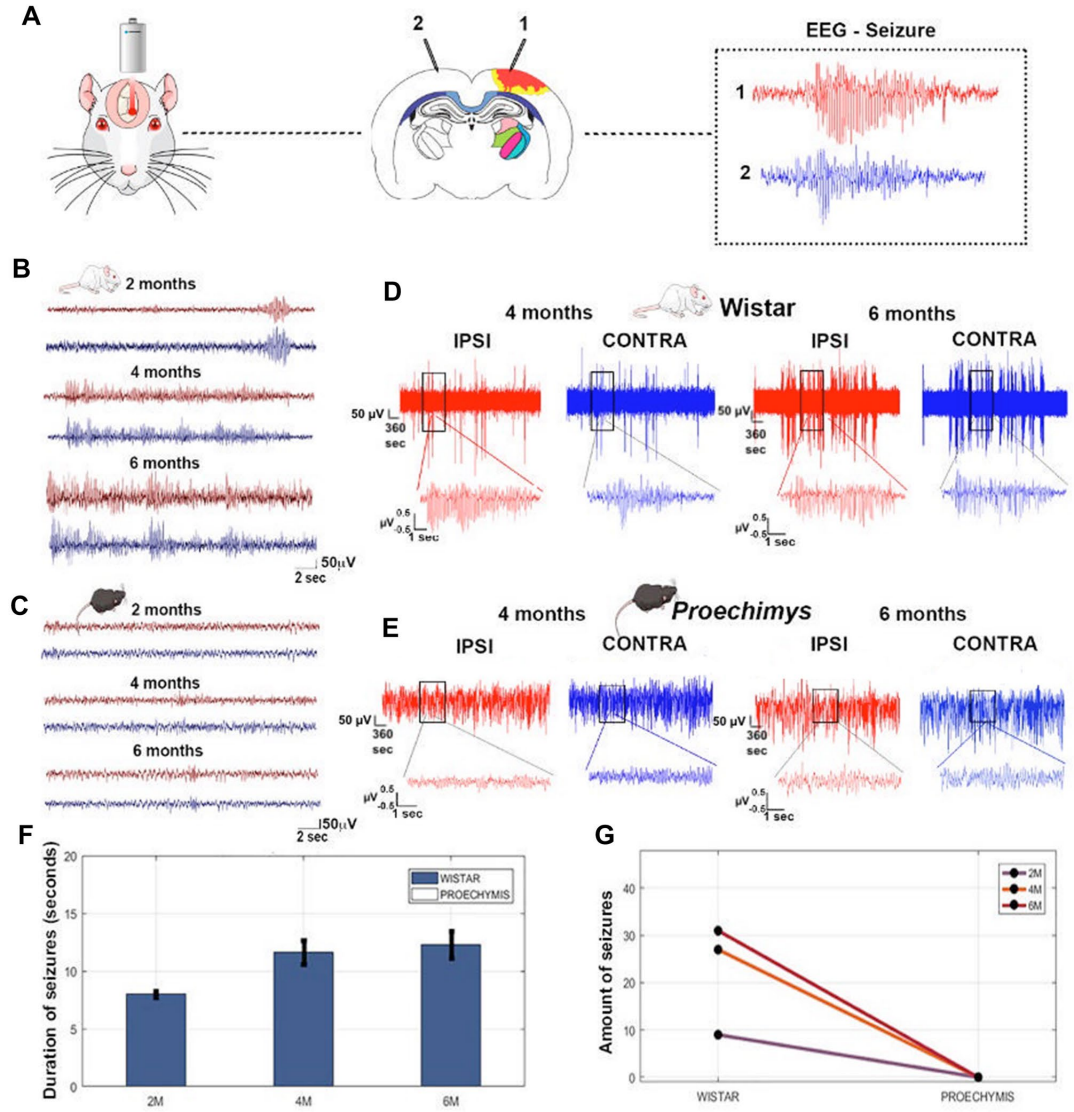
Long-term electrocorticographic monitoring after photothrombotic stroke *in Proechimys* and Wistar rats. (**A**) Schematic representation of stroke induction; the coronal brain section shows the position of the ipsilateral (1) and the contralateral (2) cortical recording electrodes; representative trace recordings of epileptiform activity are shown in the box, ipsilateral (red) and contralateral (blue) to the lesion. (**B**) Representative ECoG tracings showing epileptiform activity recorded at 2, 4 and 6 months after stroke in Wistar rats (n = 8). Note temporal changes in ECoG pattern. (**C**) Representative post-stroke ECoG recordings in *Proechimys*. The lesion does not result in epileptiform activity in this animal species (n = 8). (**D**) 1-h compacted ECoG trace from Wistar at 4 and 6 months after stroke. Note the increased recurrent epileptic burst discharges at 6 months as compared with previous ECoG recording in Wistar rats. A representative ECoG trace from a Wistar rat induced by photothrombosis showed recurrent epileptic burst discharges. (**E**) 1-h compacted ECoG trace from *Proechimys* at 4 and 6 months after stroke. No difference is observed in EcoG recording in 6 and 6 months after brain injury. A representative ECoG trait of *Proechimys* rodent after stroke showing unchanged ECoG signaling. (**D,E**) Extended view of single-cluster brain activity of the box. Time and amplitude are given by calibration 2 s—50 µV in the bottom in the ECoG recording samples. (**F**) Duration of seizures at 2, 4 and 6 months after stroke. Note that seizure activity was observed only in Wistar rats. Graphs are represented as median with 95% CI (**G**) Amount of seizures at 2, 4 and 6 months after brain injury. Graph is represented using absolute values. Figure was created in the Mind the Graph platform http://www.mindthegraph.com under Creative Commons License CC community as “attribution share-alike4.0 licensing” https://creativecommons.org/licenses/by/4.0/.

Previous studies have shown that photothrombosis injury may lead to spontaneous ictal activity ^12^. To determine whether animals will develop epileptiform activity after ischemic injury, prolonged electrocorticographic (ECoG) recordings were performed during a period of 2–6 months in Wistar and 2–12 months in *Proechimys* (Fig. 5A). Interictal and ictal recordings were obtained in 7 out of 8 injured Wistar rats (88%) (Fig. 5B,D). Conversely, none of the *Proechimys* showed spontaneous epileptiform activity during chronic monitoring for up to 12 months (Fig. 5C,E). In Wistar rats, a cortical desynchronization with low-amplitude followed by a bilateral high amplitude spike-wave activity of about 7.5 Hz lasting 8 s was found at 2 months after photothrombosis. The same pattern of ictal activity with a mean duration of 11.63 s was observed at four months evolving with a slight increase in duration at 6 months (12.29 s) following a stroke (Fig. 5B,F). Epileptiform activities manifested clinically as a motor arrest, the amount of seizure per month registered at 2, 4 and 6 months was 9, 27, 31 respectively (Fig. 5G). On the other hand, ictal activities were not observed in *Proechimys* (Fig. 5F,G)*.* The Amazon rodent showed bilaterally low amplitude desynchronized ECoG after ischemic injury (Fig. 5C). Wistar rats with photothrombotic lesions exhibited sharp-wave discharges, but control rats did not.

### Cortical spreading depression after photothrombosis reveals that Wistar rats are more affected by lesion than *Proechimys*

**Figure 6:**
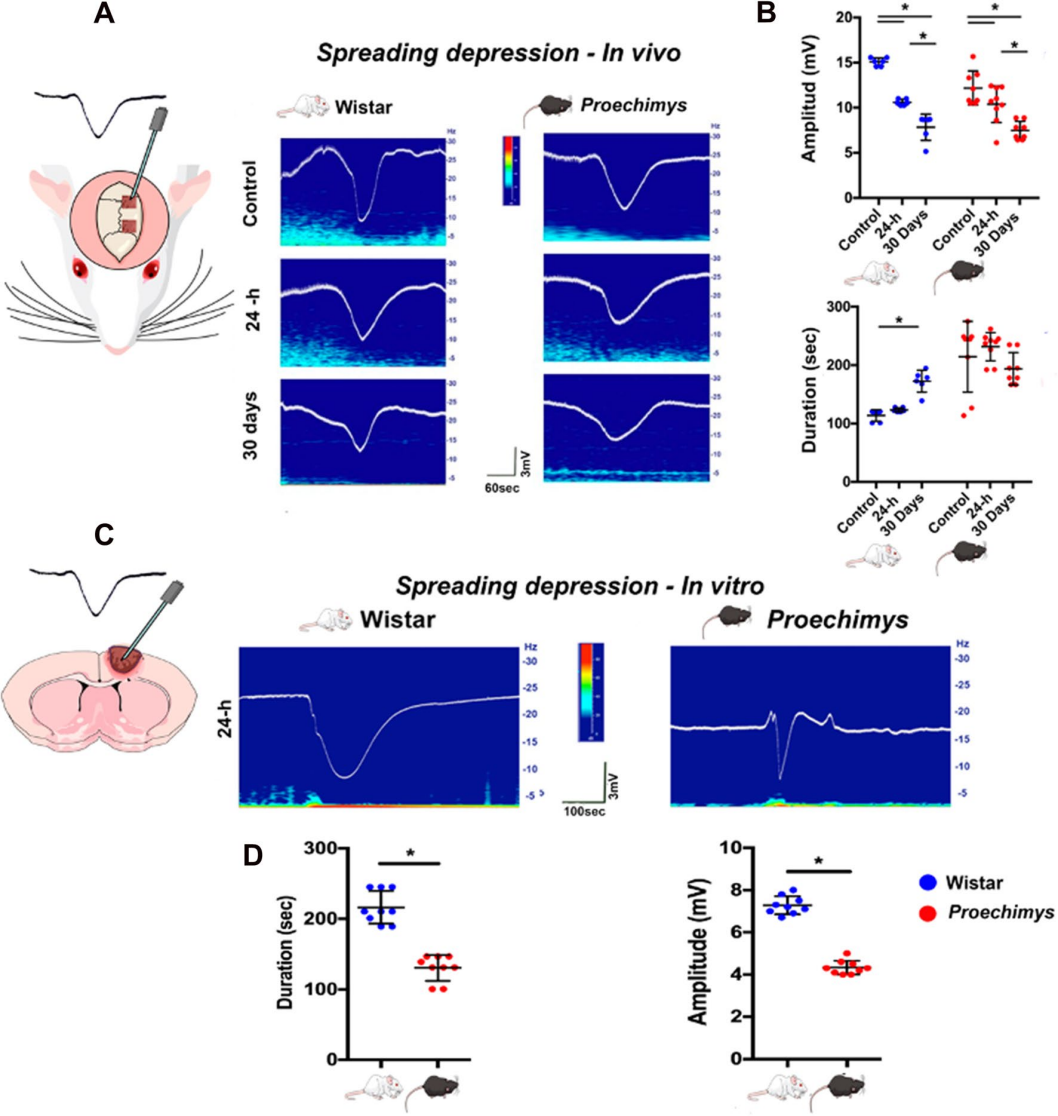
Spreading depression *recordings* in Wistar and *Proechimys*. (**A**) Representative recordings of spreading depression (SD) activity and its corresponding spectrogram in both animal species. (**B**) Statistical analysis of amplitude and duration of in vivo cortical SD recordings. In both animal species, all groups showed differences in the amplitude of SD: in Wistar (F = 98.53; p ≤ 0.0001); in *Proechimys* (F = 15.19; p ≤ 0.0001). Control *vs* 24-h, control *vs* 30 days, 24-h *vs* 30 days (Tukey’s multiple comparison test). In the duration of SD, Wistar rats showed differences between control *vs* 30 days (H = 13.24 p ≤ 0.0001) (Tukey’s multiple comparison test). Note that no differences were found in *Proechimys*. (**C**) Representative SD-like activity and its corresponding spectrogram in both animal species at 24-h after stroke. (**D**) Statistical analysis comparisons between animal species showed lower amplitude and duration of SD-like activity in *Proechimys*. Nonparametric test Mann–Whitney U = 0 *p < 0.0001. For the SD recording samples of the frequency spectrum, time and amplitude are both given by calibration bars. Graphs are represented as median with 95% CI. Figure was created in the Mind the Graph platform http://www.mindthegraph.com under Creative Commons License CC community as “attribution share-alike4.0 licensing” https://creativecommons.org/licenses/by/4.0/.

Cortical spreading depression (SD) is a phenomenon that is related to tissue excitability ^13^. From in vivo evaluation, our results showed a reduction in the amplitude in acute and long-term phases after ischemic lesion in both animal species, contrary to the non-ischemic condition in which the events of SD presented greater amplitude when compared with their respective experimental groups (Fig. 6A). These findings suggest that SD induction suffered resistance to invade the cortical injured region, which is the focus of hyperexcitability in Wistar rats, demonstrated by the spontaneous ictal discharges, or that this response could be influenced by local changes in the inhibitory mechanisms in the perilesional area, such as downregulation of GABAergic markers after stroke as observed in mice ^14^. However, Lauritzen et al. associated the duration of SD with the tissue's ability to recover after wave propagation ^15^. Therefore, longer cortical SD duration can also reflect the metabolically compromised injured brain tissue. Hence, in vitro recordings were performed in the ipsilateral cortex to the ischemic lesion to assess the incidence of SD (Fig. 6C). In these experiments, SD was induced in both animal species at 24-h after stroke, but the lower amplitude was found in *Proechimys* (4.33 ± 0.11 mV) as compared to Wistar (7.27 ± 0.14 mV) (U = 0, p < 0.0001) (Fig. 6D). Animals studied at 30 days after photothrombosis and controls did not present spontaneous SD-like activity. The duration of the SD records has been previously described and is directly associated with stroke damage size ^15^. Cortical SD is an exceptionally powerful and reproducible phenomenon in rodent models to assess brain dynamics. Changes in SD dynamics may suggest pathological activity associated with brain injury. From in vivo experiments, we observed longer SD duration in Wistar rats at 30 days after stroke (H = 13.24; p < 0.0001) when compared to both Wistar rats and control counter parts at 24-h of photothrombosis (control group = 113.8 ± 4.06 s; 24-h group = 123.3 ± 1.48 s; 30 days group = 172.7 ± 7.66 s). On the other hand, no differences were observed among the 3 animal groups of *Proechimys* rodents (H = 3.215; p < 0.2057); (control group = 214.5 ± 21.47 s; 24-h group = 231.8 ± 8.05 s; and 30 days group = 193.8 ± 14.98 s) (Fig. 6B). Longer duration of in vitro SD-like activity was found in the 24-h Wistar group after stroke (215.03 ± 7.08 s) when compared to the 24-h *Proechimys* (131.4 ± 5.53 s), (U = 0, p < 0.0001) (Fig. 6D).

## Discussion

Heterogeneities between animal species offer unparalleled opportunity to decipher disease physiopathology and distinctive host response. This work investigated the endogenous brain response elicited by photothrombosis in the *Proechimys* rodents in comparison with Wistar rats. *Proechimys* showed smaller volume of ischemic infarction and lesser degrees of glial activation than Wistar rats. Post-stroke decreased expression of pro-inflammatory cytokines and increased levels of anti-inflammatory mediators and growth factors were found in *Proechimys,* in contrast to Wistar rats. Changes in electrophysiological signaling studied by SD showed that Wistar’s brain is most severely affected by stroke. Furthermore, we found that *Proechimys* rodents are resistant to post-stroke epilepsy, suggesting endogenous antiepileptogenic mechanisms in these animals.

After ischemic stroke, expression of pro- and anti-inflammatory cytokines rapidly increases throughout the brain ^16^. Interleukin IL-1β, IL-6, IL-10 and transforming growth factor-β (TGF-β) are key mediators of damage in ischemic stroke ^17^. Inflammatory cytokines are important factors orchestrating the responses elicited by a stroke with key detrimental and protective roles. Some of these cytokines appear to exert both roles, but the reasons for this duality remain uncertain ^18^. Clinical studies showed that ischemic stroke led to increased levels of IL-1β, IL-6, and TGF-β in cerebrospinal fluid, blood, and brain tissue of patients ^19^. Treatment with IL-1β receptor antagonist or the knocking out of IL-1β receptor in mice resulted in dramatic reduction of ischemic infarct volumes ^20^. Elevated levels of IL-1β and IL-6 after cerebral ischemia are correlated with the stroke volume, severity and long-term outcome ^21^. In our study, brain levels of IL-1β and IL-6 remained unchanged after photothrombosis in *Proechimys*. However, increased levels of these cytokines were previously found in the Wistar rats ^9,22^. The present findings in *Proechimys* are consistent with what has been found in the previous study of our group that described the unchanged levels of inflammatory factors 24-h after *status epilepticus* induced by pilocarpine in *Proechimys* ^6^. Since inflammation develops rapidly after an epileptogenic insult, scientific literature has suggested the important role of the brain pro-inflammatory milieu in the development of a maladaptive process that leads to epilepsy ^23^. Here, we also found that IL-10 is acutely increased after stroke in *Proechimys*. Interleukin-10 exhibits potent anti-inflammatory properties and worse neurological outcomes are reported in patients with low IL-10 plasma levels during the first hours after stroke ^24^. IL-10 has been described to suppress IL-6 production and to counterbalance the actions of IL-1β ^25^. IL-1 also activates the endothelium to produce trophic factors such as VEGF ^26^. VEGF shows anti-inflammatory and pro-angiogenic activity and neuroprotective roles ^27^. Again, in contrast with previous studies describing post-stroke VEGF upregulation ^28^, photothrombosis did not induce an increase in VEGF levels in *Proechimys*. On the other hand, BDNF and its receptor tropomyosin-related kinase type B (TrkB) and TGF-β levels were elevated at 24-h and 30 days after stroke in *Proechimys*. On the contrary, in Wistar rats the levels of these factors were increased only at 24-h post-stroke returning to basal values after 30 days. The mechanisms inherent to *Proechimys* supports the designed clinical approaches currently used focusing on maintaining elevated levels of growth factors after brain injury as a reparative strategy to control the inflammation associated with stroke ^29^. BDNF has been shown to play a role in both protection and recovery of functions after stroke, exerting anti-inflammatory, anti-apoptotic effects, among others ^30^. Acutely elevated levels of TGF-β1 in the serum of patients were correlated with better clinical outcomes 1 month after ischemic stroke ^31^. While many studies report the beneficial effects of TGF-β1 after ischemic stroke, its roles in worsening the injury have been described as a key mediator in brain injury ^32,33^. TGFβ-signaling in astrocytes has been implicated in post-stroke epilepsy ^17,33^. In line, therapeutic approaches to inhibit TGF-β have been suggested to prevent post-stroke epilepsy ^34^. On the other hand, rodents with deficient astrocytic TGF-β signaling exhibited greater brain inflammation following photothrombosis than wild-type animals and late infarct expansion^35^. Several inflammatory and trophic factors have been proposed as strategies for intervention after brain injury ^36^. However, taking into consideration the complexity of stroke physiopathology, it seems that working on a set of growth factors and anti-inflammatory modulators would improve the chances of achieving favorable restorative outcomes ^37^. Following ischemic stroke, activated microglia and astrocytes in the damaged area release a variety of inflammatory mediators and growth factors among other factors, making these glial cells major targets for intervention to minimize the burden of stroke ^38^. Stroke is known to induce a prompt activation of microglia ^39^, nevertheless, a major finding of our work is the absence of activated microglia after photothrombosis in *Proechimys* rodents in contrast with the robust activation detected in Wistar rats, which also exhibited abundant proliferating microglia. It has been reported that microgliosis is eminently formed by proliferation of resident parenchymal microglia after photothrombosis ^40^. Microglia is a crucial immune system cell to defend an injured-brain and the brain innate immune response to injury is orchestrated mainly by its resident microglial and astrocytic populations ^41^. *Proechimys* rodents inhabit Amazon rainforest, a major endemic area for many tropical diseases, and these rodents have attracted interest from biomedical research as key reservoirs of zoonotic viruses and parasites, as well as their natural resistance to tropical diseases ^8^. Perhaps the intrinsic immune responses modulated by the environmental factors of the natural habitat in which these animals evolved play a role in brain glial activation and expression of cytokines and inflammatory mediators after brain injury. According to our study, brain lesion induced an abundant delayed activation of astrocytes and neuronal loss in the border infarct area and ipsilateral thalamic nuclei in the Wistar rats, whereas *Proechimys*’s thalamus was much less affected after a stroke. Complex network interconnected brain regions are reorganized after pathological perturbations, but little is known about how maladaptive thalamocortical system reorganization affects recovery from ischemic stroke^42^. Previous studies have reported that photothrombosis promote thalamocortical system remodeling associated with focal gliosis and neuronal loss leading to hyperexcitation after stroke^11,43^. These authors showed that silencing thalamic regions of interest through optogenetic approaches is sufficient to stop epileptic seizures in post-stroke epilepsy scenario^11^. Currently, approaches by targeting brain connections to manipulate cortical activity and excitability are showing promise for their potential for improvement of post-stroke outcomes^42^. Depressed evoked activity is registered in the ipsilateral cortex for weeks following stroke^44^. Cortical spreading depression (SD) is an excitability-related neural response and SD velocity of propagation is a reasonable way of estimating cortical susceptibility to SD ^45^. Cortical stroke, associated with a multifactorial set of deleterious alterations, impaired the velocity of SD propagation in both animal species but Wistar rats showed slower SD propagation velocity than *Proechimys* suggesting that ischemic infarct has a greater impact on Wistar’s brain. Lower rates of SD propagation are suggestive of higher resistance exhibited by the cortical tissue to the phenomenon ^46^. Nevertheless, the epileptic brain undergoes excessive tissue excitability and becomes more resistant to SD ^47^. ECoG recording was registered in both animal species. In Wistar rats, epileptiform activity was observed starting at 2 months after photothrombotic lesion and it was maintained present during the 6 months assessed. However, in Proechimys rodents, after 6 months of ECoG recordings, epileptiform activity was not observed. In order to allow for the Proechimys’ brain to manifest electrical activity disturbs, ECoG was performed extended for 12 months. Since epileptiform activity was observed in Wistar rats during the months evaluated, no prolonged ECoG was necessary for this group, despite prolonged monitoring, epileptiform activity was not observed in Proechimys. Although stroke represents a common cause of epilepsy, the complex mechanisms underlying epileptogenesis remain obscure. Photothrombotic stroke has been recognized and accepted as a model for poststroke epilepsy in rodents ^12,48^. The focal cortical ischemic lesion induced by rose Bengal photothrombosis leads to post-injury epilepsy in approximately 50% of the rodents ^11^. In our work, photothrombosis resulted in spontaneous ictal activity in 88% of the Wistar rats. Our major finding is that *Proechimys* rodents are resistant to epileptogenesis since none of the animals developed spontaneous seizures, suggesting endogenous antiepileptogenic mechanisms in these animals. Brain responses to injury seem to be an intrinsic organ capacity. However, stroke-induced reorganization can be either beneficial, trivial or even maladaptive for recovery. In *Proechimys*, brain responses after photothrombotic stroke appear to drive the favorable routes. Dissimilarities between animal species offer unparalleled opportunity to decipher disease pathophysiology and distinctive host response, and fortuitously find novel disease-modifying therapies. Here, *Proechimys* and Wistar rats showed different brain responses to stroke. The evolutionary history of the animal species and the selective pressures imposed by the environment are both thought to play key roles in the development and progression of diseases. Our study on the responses of the *Proechimys*´s brain to ischemic stroke has yielded findings suggesting that these Amazon rodents may serve as an animal model to gain insight into antiepileptogenic mechanisms after potentially epileptogenic brain insults.

## Methods

### Study design

The aims of the present study were to assess the multifaceted brain responses to photothrombotic cortical ischemia in the Amazon rodent *Proechimys* in comparison with Wistar rats. Comparative approaches between these two animal species were used at 24-h and 30 days after stroke.

All experimental procedures were approved by the Universidade Federal de São Paulo (UNIFESP) Ethical Committee (CEUA 4124210916) and were carried out in accordance with the National Health Institute guide for care and use of laboratory animals and under permission of the Brazilian Institute for the Use and Conservation of the Environment (IBAMA, registration 1561643). The experiments followed the ARRIVE (Animal Research: Reporting of In Vivo Experiments) guidelines. Male Wistar rats and wild-derived Proechimys rodents (200–300 g) were used for all experiments. Animals were housed under environmentally controlled conditions (22 °C ± 1 °C), 12/12 h light–dark cycle and free access to water and food.

### Induction of cortical ischemia by photothrombosis

Focal infarction was performed in cortical vasculature according to the protocol of Watson et al. (1985). Wistar rats were anesthetized intraperitoneally with ketamine-xylazine (1.6 and 0.6 mg/kg, respectively) and *Proechimys* rodents with Ketamine and xylazine (1.8 and 1.2 mg/kg, respectively). The animal temperature was monitored with a rectal thermometer and kept at 36.5 ± 0.5 °C with a hot pad. Rose Bengal solution (20 mg/kg) was administered through inguinal access of the left femoral vein. A longitudinal incision in the scalp of the animal was performed to expose the bregma and to allow a 15-min application of halogen cold light (diameter of 4.5 mm) on a spot on the left parietal bone 1 mm posteriorly and 1 mm laterally to the Bregma to induce an ischemic vascular lesion ^49,50^. After the procedure the animal was sutured, left to recover from anesthesia and returned to the animals’ facility. A subset of animals received the Rose Bengal solution only, and another group of animals solely received halogen cold light application but neither approach elicited brain lesion.

### Ischemic lesion volume

Ischemic infarct size was assessed at 24-h and 30 days after photothrombosis using coronal sections stained with Cresyl violet acetate (Sigma Aldrich, USA). To perform volume estimation using cresyl violet stained sections, the rodents were perfused with 4% paraformaldehyde solution, the brains were removed and post-fixed overnight at 4 °C. Then brains were sliced into 40 µm sections and stained in cresyl violet. One slice was selected every 5 section (200 μm) therefore, thirty sections were analyzed per animal in both animal species. This sampling allows us to estimate the volume of the ischemic lesion with precision. Image of each Nissl-stained section was digitized, and the infarct size was delimited and measured with the Stereo Investigator software according to the Cavalieri principle. The ischemic infarct size of the slices was determined and multiplied by the mean section thickness and shown as a percentage of the intact ipsilateral hemisphere. For these experiments, five rats of each animal species were used.

### Immunofluorescence

Rodents (control, 24-h and 30 days after stroke) were transcardially perfused with phosphate-buffered saline followed by 2% paraformaldehyde. The brains were removed after perfusion and post-fixed in 2% paraformaldehyde for 4-h. Brains were sectioned in 40 µm thickness using a vibratome (Leica VT1200S) and sections in rostral-caudal direction, from 1.6 to − 3.6 mm, using the bregma as reference were selected. Brain sections were treated with PBS containing 10% of bovine albumin and 0.3% Triton X-100 for 4-h at 4 °C, and then incubated for 48-h with the following primary antibodies: CD11-b clone [OX-42] (mouse monoclonal antibody; 1:100; Abcam) and glial-fibrillary-acidic-protein anti-GFAP (rabbit polyclonal antibody; 1:1000; Abcam). Next, sections were incubated for 2-h at room temperature in the dark with the secondary antibodies anti-IgG rabbit (goat polyclonal conjugated with DyLight 488; 1:250, Abcam) and anti-IgG mouse (goat polyclonal conjugated with DyLight 594, 1:250; Abcam). Sections were sealed under coverslips using glycerol and analyzed by a fluorescence microscope. The quantitative analysis of the intensity of the CD11-b and GFAP immunoreactivity was performed in terms of the percentage of optical absorption (POA). Photomicrographs, obtained from 2 to 4 sections per animal, with about 200 µm between sections, were obtained using a confocal microscope (Zeiss LSM 710) equipped with a primary beam splitter of 488/543, using an argon laser of 488 nm for the secondary antibody conjugated to the fluorophore Dylight 488, and a helium–neon laser of 543 nm for the secondary antibody conjugated to the fluorophore Dylight 594. The images were captured using a 10× lens for the complete reconstruction of the brain slice, producing an image of approximately 13,000 × 8000 pixels (104 megapixels). By means of this high-resolution photomicrograph, the brain regions most affected by the ischemic injury and those with marked glial immunoreactivity were identified and were then captured with a 63× glycerol immersion lens (63-Plan/Apochromat 63×/1.40 OilDic M27) for optical densitometry analysis. The pinhole was configured for both magnifications in 1 Airy unit.

Optical densitometry analysis was performed to assess the intensity of CD11-b and GFAP immunoreactivity. The photomicrographs obtained through the 63× objective (225 µm^2^; 2–5 per region of interest) were sampled in both hemispheres (ipsilateral and contralateral to the lesion). Confocal photomicrographs were processed in RGB and compressed to gray scale (RGB meanband), in order to obtain the corresponding histograms. To improve contrast, a histogram equalization technique was incorporated into the analysis ^51^. In addition, to avoid interference of neuronal lipofuscin autofluorescence, its spectrum was digitally subtracted from the samples of each photomicrograph collected. The resulting images provided an intensification of the pixels corresponding to the immunostaining, which resulted in more reliable segmentations. A gray scale interval (between 0 and 65 in gray scale) was adopted as a limit to consider the immunoreactive area of the tissue. The significant pixels were later converted into a binary matrix (black and white) and quantified by the sum of black pixels per area. Quantification was performed using a computer system developed in MATLAB, with photomicrographs showing high resolution and the same optical zoom. The data were plotted as a percentage of equivalent immunoreactivity for each photomicrograph. For cells immunostaining (GFAP and CD-11), each animal species was divided respectively as follows: control (n = 4); 24-h (n = 4) and 30-day (n = 5).

### Multiplex bead array assay (MBAA) of cytokines and growth factors

MBAA was used to assess the brain levels of inflammatory cytokines and growth factor in the different animal groups (control, 24-h and 30 days after stroke) of both species. Immunoassay measurements were performed according to the manufacturers’ guidelines (Milliplex/Millipore-cytokine/chemokine magnetic bead panel RECYTMAG-65K-05; CYT306; TGFBMAG-64K-01; E-EL-R1046 and E-EL-R1430) for the following cytokines/chemokines: IL-1β, IL-6, IL-10, TGF-β, VGEF, BDNF, TrkB. Briefly, the assay uses 25 µL of sample to analyze the cytokines/chemokines previously mentioned. Then detection with antibodies were performed followed by an incubation with specific solution. All measures were performed using Luminex xPonent 4.2 software.

To perform these experiments, the hemispheres were homogenized and treated as previously described ^6^. Briefly, the animals were anesthetized with thiopental, 80 mg/kg, i.p., then decapitated and brains were rapidly removed for posterior dissected on ice. Brain tissue was rapidly placed in ice-cold homogenization buffer solution. Brain tissues were homogenized, boiled, and centrifuged at 4 °C. After that, supernatants were collected and stored at − 80 °C until use. The number of animals used to determine IL-1β levels in Wistar groups, control, n = 12; 24-h, n = 11; 30 days, n = 12) and *Proechimys* groups, control, n = 11; 24-h, n = 11; 30 days, n = 12. For IL-6 levels Wistar groups: control, n = 10; 24-h, n = 12; 30 days, n = 11 and *Proechimys* groups: control, n = 10; 24-h, n = 10; 30 days, n = 12). For IL-10 levels, Wistar groups: control, n = 11; 24-h, n = 12; 30 days, n = 11) and *Proechimys* groups: control, n = 10; 24-h, n = 10; 30 days, n = 9. For TGF-β levels, Wistar groups: control, n = 12; 24-h, n = 11; 30 days, n = 12 and *Proechimys* groups, control, n = 9; 24-h, n = 11; 30 days, n = 9. For BDNF levels, Wistar groups, control, n = 11; 24-h, n = 12; 30 days, n = 10 and *Proechimys* groups: control, n = 12; 24-h, n = 12; 30 days, n = 10. For TrkB levels Wistar groups: control, n = 12; 24-h, n = 12; 30 days, n = 11 and *Proechimys* groups: control, 24-h, n = 11; 30 days, n = 10. For VEGF levels Wistar groups: control, n = 12; 24-h, n = 10; 30 days, n = 11 and *Proechimys* groups, control, n = 11; 24-h, n = 11; 30 days, n = 10.

### Cortical spreading depression (SD) in vivo

The procedures were performed with animals anesthetized with intraperitoneal injection of 10 ml/kg of a solution of 100 mg urethane and 40 mg alpha chloralose (Sigma Aldrich, USA) diluted in 100 ml of saline solution. Rodents were divided into the following groups: control, 24-h and 30 days. Two windows with 2 to 3 mm size were opened in the skull, one centrally to the frontal bone at the level of the animal’s eyes for the induction of the SD and the second in the left parietal bone at the same coordinates of the brain injury (1 mm posteriorly and 1 mm laterally to the bregma) for signal recording. Silver/silver chloride (Ag/AgCl) electrode was placed on the meninges exposed above the lesion and the ground electrode set on the nasal bone of the animal. SD was induced by leaving a 1 to 2-mm cotton ball soaked with 2% potassium chloride (270 mM) on the meninges exposed in the frontal bone window for 1 min. A total of 5 inductions were performed in intervals of 20 min. Frequencies below 3 kHz and above 10 kHz were filtered for the recording, which were acquired and digitalized by a micro CED-1401 data acquisition unit (Cambridge—UK) and analyzed on Spike2 software, version 6.09. Animals were kept at 37 ± 1 °C ^52^ during all the procedures. To perform these experiments, the number of animals used per animal species was, in Wistar groups: control n = 6; 24-h, n = 6; 30 days, n = 6; in *Proechimys* groups: control n = 8; 24-h, n = 8; 30 days, n = 9.

### Long-term intracranial electroencephalography

Two months after stroke, Wistar rats and *Proechimys* rodents had micro-wire electrodes implanted for long-term electrographic recordings as previously described ^53^. Briefly, after exposure of the skull surface, two small holes in the frontal and parietal bones at each hemisphere were drilled in close proximity to the bregma. The following coordinates were established in both animal species as follows; reference electrode in left and right frontal cortex (AP: + 2.5 mm, ML: − 1.3 mm, relative to bregma); recording electrode in left and right parietal cortex (AP: − 2.2 mm, ML: − 2.5 mm. (AP: + 2.5 mm, ML: + 1.3 mm). The reference electrode was anchored in the nasal bone (AP: + 2.5 mm, ML: + 1.3 mm, relative to the bregma).

Electrodes were made from a 150 µm diameter nickel–chromium wire. All four electrodes were previously welded to an individual pin socket of a micro-connector, which was posteriorly fixed to the animal’s skull with methyl methacrylate resin after positioned in the coordinates previously described. Surgical procedures were performed under anesthesia and animals were left to recover 7 days before monitoring. The electroencephalographic recording was performed on awaked animal placed in a Faraday cage during 5-h per day in the morning throughout 12 months with signals recorded at 1000 Hz and analyzed using Spike2 software version 6.09.

Interictal epileptiform activity was defined as paroxysmal electrical sharp activity with duration between 20 and 150 ms and voltage that is 5 times the background electroencephalographic activity. Electrographic seizure was defined as repetitive epileptiform electroencephalographic discharges at more than two cycles per second and/or characteristic pattern with quasi-rhythmic spatio-temporal evolution (gradual change in frequency, amplitude, morphology and location), lasting at least several seconds (> 10 s). For seizure detection, a manual review was performed by two investigators in a double-blinded manner. To perform these experiments, 8 rats in each animal species were used.

### Cortical spreading depression in vitro

At 24-h and 30 days after photothrombotic stroke, a subset of animals were anesthetized with isoflurane at 1% dissolved in 70% N_2_O and 30% O_2_. Then, brains were removed quickly and sections of 400 μm thickness were prepared for electrophysiological recording as previously described ^54^. The recording electrode (chlorinated silver wire in glass capillary filled with sodium chloride 154 mM; 2–4 MΩ) was positioned in the cortical boundary zone of stroke. Spreading depression assay was performed as described by Lapilover et al. The assay started immediately after removing albumin from the interface chamber and continued for 30 min. Slices exhibiting population spikes with a maximum of 1.5 mV of the beginning of the assay or loss greater than 20% of its maximal amplitude value were excluded from the analysis. Frequencies below 3 kHz and above 10 kHz were filtered out for the recording of signals. In these experiments, 3 animals per group were used and 3 slices per animal were investigated (Wistar, animals/slices n = 3/9; Proechimys, animals/slices n = 3/9).

### Statistical analysis

Shapiro–Wilk test was applied to verify normality distribution of the data and to select a correspondent parametric or nonparametric test. For two-sample comparisons, Student’s T-test or Wilcoxon (for non-parametric data model) was used. For group comparison, analysis of variance (ANOVA-one way) or its non-parametric version, the Kruskal–Wallis test was applied followed by Tukey post hoc test for multiple comparisons. Level of statistical significance was set to α = 0.05. Data are expressed as the mean ± SD for parametric test and median ± 95% CI for non-parametric tests, where SD = standard deviation and CI = confident interval. The software SPSS 25.0 (Chicago IL, USA) was used for the statistical analysis.
